# Coadministration of indomethacin and minocycline attenuates established paclitaxel-induced neuropathic thermal hyperalgesia: Involvement of cannabinoid CB_1_ receptors

**DOI:** 10.1038/srep10541

**Published:** 2015-06-18

**Authors:** Subramanian S. Parvathy, Willias Masocha

**Affiliations:** 1 Department of Pharmacology and Therapeutics, Faculty of Pharmacy, Kuwait University, Kuwait

## Abstract

Taxanes such as paclitaxel, which are chemotherapeutic drugs, cause dose-dependent painful neuropathy in some patients. We investigated whether coadministration of minocycline and indomethacin produces antinociceptive effects in mice with paclitaxel-induced neuropathic thermal hyperalgesia and if the cannabinoid system is involved. Previously, we reported that coadministration of these two drugs results in antinociception against inflammatory pain at doses where either drug alone lack significant activity. In the current study, we observed that treatment of female mice with indomethacin or minocycline alone did not affect established paclitaxel-induced thermal hyperalgesia, whereas coadministration of the two drugs attenuated it. In male mice indomethacin had some antihyperalgesic activity, whilst minocycline did not. Coadministration of the two drugs had supraadditive antihyperalgesic activity in male mice. Administration of a cannabinoid CB1 receptor antagonist AM 251 blocked the antihyperalgesic effects of the combination of minocycline and indomethacin in both male and female mice. In conclusion our results indicate that coadministration of minocycline and indomethacin abrogates established paclitaxel-induced neuropathic thermal hyperalgesia in mice, and the potentiation of the antinociceptive effects of this combination involves the cannabinoid system.

Combinations of two or more drugs are used frequently to alleviate either acute or chronic pain. The combinations often achieve more analgesic activity and fewer side effects, utilising less dose of each drug in the combination, compared to each drug being used alone^1^,^2^,^3^,^4^. There is a need for synergistic multimodal analgesia using drugs with different mechanisms of action because of the complexity of the neurobiology of different types of pain and the inability of unimodal analgesia to alleviate various types of pain, including neuropathic pain. In previous studies, we observed that the combination of minocycline with indomethacin had more antinociceptive effects against thermal pain and lipopolysaccharide-induced thermal hyperalgesia and arthritis than when either drug was used alone^5^. These findings suggested that lower doses of indomethacin could be used in combination with minocycline to treat pain. The use of lower doses of indomethacin would reduce dose-dependent side effects such as gastrointestinal bleeding caused by indomethacin and other nonsteroidal anti-inflammatory drugs (NSAIDs)^6^. Indomethacin is a NSAID, whereas minocycline is a tetracycline antibiotic with anti-inflammatory activities. Both indomethacin and minocycline have anti-inflammatory activities and they are both used in the management of inflammatory diseases such as rheumatoid arthritis^7^,^8^,^9^.

Previously, we hypothesized that the enhanced antinociceptive activities of coadministration of indomethacin and minocycline could be partly due to their effects on the endocannabinoid system^5^, since each drug differentially modulate the endocannabinoid system and this has been associated with their antinociceptive activity^10^,^11^. Cannabinoid receptor agonists prevent or suppress paclitaxel-induced neuropathic pain^12^,^13^,^14^. The development of dose-limiting neuropathy, which for many results in neuropathic pain, limits the use of paclitaxel as a chemotherapeutic drug against solid tumours such as breast and ovarian cancer^15^,^16^. Thus, we evaluated the effects of the coadministration of minocycline and indomethacin in a mouse model of paclitaxel-induced neuropathic pain^17^,^18^ and whether the CB1 receptors are involved in the activity of the combination.

## Results

Effects of treatment with indomethacin and minocycline alone or in combination on female and male mice with paclitaxel-induced thermal hyperalgesia

**Figure 1:**
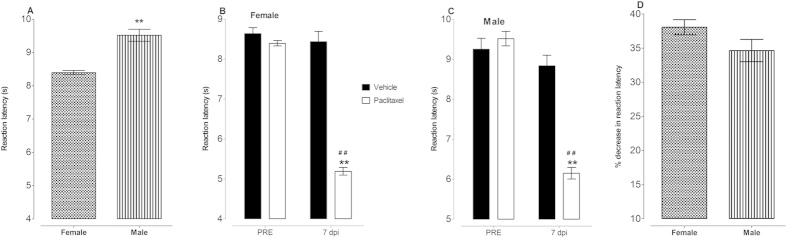
Reaction latency to thermal nociception and paclitaxel-induced thermal hyperalgesia in female and male BALB/c mice. (**A**) Baseline reaction latency times taken in hot plate test before administration of paclitaxel (n = 48 female mice and 52 male mice) **p < 0.01 compared to female mice. Thermal hyperalgesia in (**B**) female mice and (**C**) male mice at day 7 post first inoculation of paclitaxel (administered at 2 mg/kg, i.p. for 5 consecutive days; for females n = 8 vehicle-treated and 48 paclitaxel-treated mice and for males n = 10 vehicle-treated and 52 paclitaxel-treated animals). **p < 0.01 compared to drug vehicle at the same day after treatment and ##p < 0.01 compared to pretreatment (PRE) values. (**D**) Percentage decrease in reaction latency times (hyperalgesia at day 7 post first inoculation of paclitaxel, administered at 2 mg/kg, i.p. for 5 consecutive days; n = 48 female mice and 52 male mice).

Unpaired Student’s t test showed that the baseline reaction latency to the hot plate test was higher in male mice compared to female mice, 9.5 ± 0.2 s versus 8.4 ± 0.1 s, respectively (p < 0.0001; n = 48 for female mice and n = 52 for male mice; Fig. 1A) Paclitaxel-treated female and male mice had thermal hyperalgesia on day 7 after first drug administration. Unpaired Student’s t test showed that administration of paclitaxel produced a significant reduction in the reaction latency times (thermal hyperalgesia) on day 7 after first drug administration from 8.4 ± 0.1 s to 5.2 ± 0.1 s in female mice and from 9.5 ± 0.2 s to 6.2 ± 0.1 s in male mice compared to baseline (pretreatment) values (p < 0.0001; n = 48 for female mice and n = 52 for male mice; Fig. 1B and C). Two-way repeated measures ANOVA showed that mice treated with paclitaxel had similar baseline values but lower reaction latency at day 7 post administration compared to vehicle-treated animals (p > 0.05 and p < 0.001, respectively; Fig. 1A and B). Unpaired Student’s t test showed that there was no significant difference in the effect of paclitaxel in the reduction in response latency time to thermal stimuli between female and male mice (p > 0.05); 38.1 ± 1.1% female mice versus 34.7 ± 1.6% for male mice (Fig. 1D).

**Figure 2:**
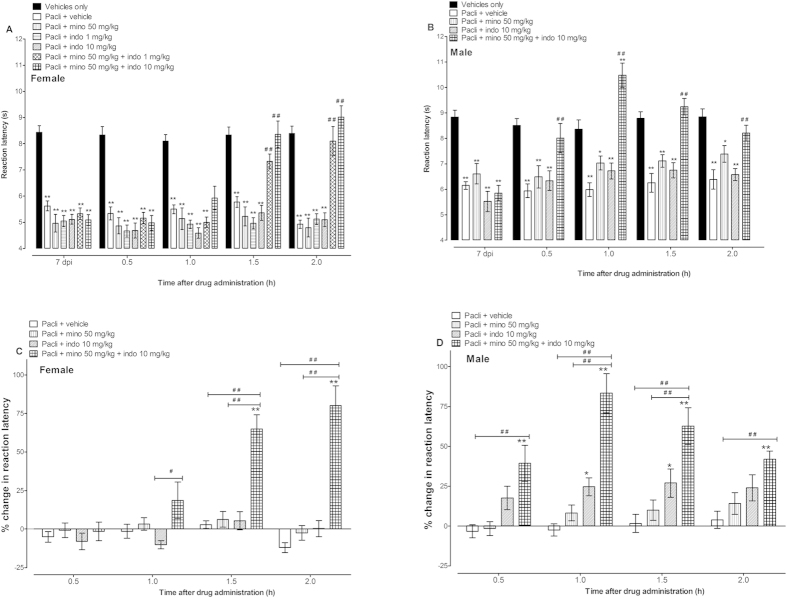
Effects of minocycline and indomethacin alone or in combination on paclitaxel-induced thermal hyperalgesia in female and male BALB/c mice. Reaction latency times of (**A**) female mice and (**B**) male mice (taken at day 7 post first administration of paclitaxel [pacli]) at different times after treatment with minocycline (mino, 50 mg/kg), indomethacin (indo, 1 or 10 mg/kg) alone or in combination in the hot plate test (n = 8 for female mice and n = 10-11 for male mice). *p < 0.05 and **p < 0.01 compared to vehicle only group (without hyperalgesia) at the same time point after treatment and ##p < 0.01 compared to mice with paclitaxel-induced hyperalgesia treated with vehicle. Percentage change in reaction latency times from baseline values (taken at day 7 post first administration of paclitaxel) of (**C**) female mice and (**D**) male mice after treatment with minocycline, indomethacin alone or in combination in the hot plate test. *p < 0.05 and **p < 0.01 compared to paclitaxel + vehicle-treated group at the same time point after treatment and ##p < 0.01 between mice treated with minocycline or indomethacin alone and those treated with minocycline + indomethacin.

Two-way repeated measures ANOVA showed that the administration of indomethacin (1 or 10 mg/kg) or minocycline (50 mg/kg) to female mice with established paclitaxel-induced thermal hyperalgesia did not produce significant changes compared to vehicle treated mice (p > 0.05; n = 8 for all groups; Fig. 2A and C). Whereas, indomethacin (10 mg/kg), but not minocycline, had significant antihyperalgesic activity in male mice with established paclitaxel-induced thermal hyperalgesia (mice (p < 0.05; n = 9–11 per group; Fig. 2B and D). Coadministration of indomethacin with minocycline significantly increased the percentage change, from baseline values, in reaction latency in both female and male mice with paclitaxel-induced thermal hyperalgesia compared to vehicle-treated animals and animals treated with either drug alone (p < 0.01; Fig. 2C and D) and completely attenuated paclitaxel-induced thermal hyperalgesia i.e. reaction latency values similar to control mice without thermal hyperalgesia (p > 0.05; Fig. 2A and B). The effect of the combination of minocycline and indomethacin was time and sex dependent; from the time of administration it produced significant effects from 0.5 h for male mice and from 1.5 h onwards for female mice (Fig. 2A–D).

Effects of a cannabinoid CB1 receptor antagonist AM 251 on the antihyperalgesic activities of the combination of indomethacin and minocycline in female and male mice with paclitaxel-induced thermal hyperalgesia

**Figure 3:**
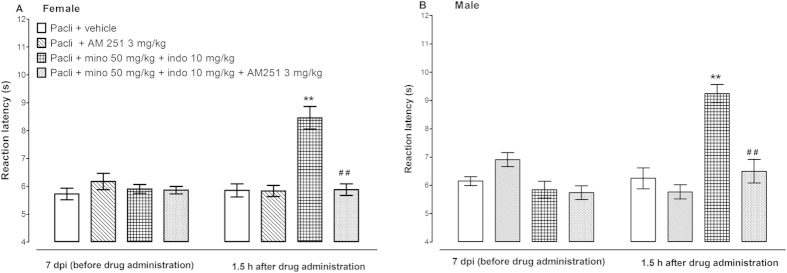
Effects of a CB1 receptor antagonist AM 251 on the antihyperalgesic effects of the combination of minocycline (mino, 50 mg/kg), indomethacin (indo, 10 mg/kg) in female and male BALB/c mice with paclitaxel (pacli)-induced thermal hyperalgesia (n = 13–17 for female mice and n = 10–11 for male mice). **p < 0.01 compared to mice with paclitaxel-induced hyperalgesia treated with vehicle at the same time point after treatment and ##p < 0.01 between mice with paclitaxel-induced hyperalgesia treated with minocycline + indomethacin and those treated with minocycline + indomethacin + AM 251.

Two-way repeated measures ANOVA showed that the administration of the cannabinoid CB1 receptor antagonist AM 251 (3 mg/kg) to both female and male mice with paclitaxel-induced thermal hyperalgesia did not alter the reaction latency to hot-plate test compared to vehicle-treated mice at 1.5 h post treatment (p > 0.05; Fig. 2). However, AM 251 significantly antagonized the antihyperalgesic effect of the combination of indomethacin and minocycline in both female and male mice, i.e. a 30% reduction in reaction latency, 8.5 ± 0.4 s for indomethacin + minocycline compared to 5.9 ± 0.2 s for indomethacin + minocycline + AM 251 for female mice and 30% reduction in reaction latency, 9.3 ± 0.3 s for indomethacin + minocycline compared to 6.5 ± 0.4 s for indomethacin + minocycline + AM 251 for male mice (p < 0.001; Fig. 3).

## Discussion

Recently, we reported that coadministering indomethacin and minocycline resulted in enhanced antinociceptive activity against inflammatory pain in arthritic mice^5^. The current study shows that the combination has supraadditive antihyperalgesic activity and abrogates paclitaxel-induced neuropathic thermal hyperalgesia, and this enhanced activity is possibly through the cannabinoid system since it is antagonized by a cannabinoid CB1 receptor antagonist.

The ability of the combination of indomethacin and minocycline to attenuate paclitaxel-induced thermal hyperalgesia could make this combination useful for managing symptoms of painful neuropathy in patients undergoing chemotherapy with a regimen containing paclitaxel. Painful neuropathy is one of the dose-limiting side effects of paclitaxel, which hamper its use in the management of solid tumors such as breast and ovarian cancer^15^,^16^. Currently, only duloxetine has a moderate recommendation for the management of chemotherapy-induced painful neuropathy (CIPN), whilst other drugs used for other neuropathic pain conditions may be given because of the limited CIPN treatment options^19^. Thus, there is a need for new drugs or the use of different drugs with synergistic effects to manage paclitaxel-induced neuropathic pain and CIPN in general.

Minocycline is a second-generation semisynthetic tetracycline antibiotic that has pleiotropic biologic activities and is used in the management of various inflammatory diseases including rheumatoid arthritis, apart from its use as an antibiotic^9^. It has been reported that preemptive treatment with minocycline in animal models of neuropathic pain has protective effects^10^,^20^,^21^,^22^,^23^ but is ineffective once pain has developed^22^,^23^. Similarly, in the current study treatment with minocycline alone was ineffective in attenuating existing paclitaxel-induced thermal hyperalgesia.

Indomethacin is a NSAID used in the management of various inflammatory diseases such as rheumatoid arthritis, osteoarthritis, gout, and ankylosing spondylitis^7^,^24^,^25^,^26^. Various studies have reported that indomethacin was inactive against neuropathic pain^27^,^28^. In the current study indomethacin lacked activity against paclitaxel-induced thermal hyperalgesia in female mice but had some activity against the hyperalgesia in male mice. Our findings concur with those of other groups that reported sex differences in response of humans to the analgesic activities of NSAIDs, where some NSAIDs were found to be ineffective against pain in females whilst they were effective in males^29^,^30^,^31^.

This is the first study to report that coadministering indomethacin and minocycline, at doses where the individual drugs had partial or no activity, produces supraadditive effects and completely abrogate established paclitaxel-induced thermal hyperalgesia. The effects of the combination were immediate (0.5 h after administration in males and 1.5 h in females) suggesting that this was possible due to inhibition of enzyme activity. Indomethacin inhibits cyclooxygenase (COX) activity and this partly is responsible for its antinociceptive activity. Minocycline inhibits the activity of lipoxygenases (LOXs)^32^, which could partly be responsible for its limited activity. Combination of COX inhibitors with LOX inhibitors have been shown to have synergistic antinociceptive activity^33^.

Both indomethacin and minocycline independently modulate the levels of endocannabinoids^10^,^11^ and thus increased levels of endocannabinoids could also be responsible for their enhanced activity against paclitaxel-induced neuropathic pain. The hypothesis that the combination of indomethacin and minocycline might work via the endocannabinoid system is further strengthened by our observation that the enhanced antihyperalgesic activity of the combination is blocked by a cannabinoid CB1 receptor antagonist.

One of the possible advantages of the use of the combinations of minocycline and indomethacin or another NSAID over the use cannabinoids is the fact that minocycline, indomethacin or other NSAIDs are already being used clinically for management/alleviation of pain and inflammation and their clinical parameters including side effects profile are well known. While on the other hand, cannabinoids are still to be approved for the management of pain. Another advantage of the combination is the multimodal analgesia based on various different mechanisms of action of the individual drugs, which is important taking into consideration the complex dynamic nature of CIPN, which so far has rendered unimodal analgesia insufficient to manage it. The combination of minocycline with indomethacin or other NSAIDs could also allow the use of lower doses of NSAIDs to manage pain, which could reduce the dose-limiting side effects of NSAIDs such as gastric bleeding.

In conclusion, using a mouse model of paclitaxel-induced neuropathic pain we show that coadministration of indomethacin with minocycline potentiates their effects and results in complete abrogation of thermal hyperalgesia where either drug alone has partial or no significant activity. The enhanced activity of the combination is partly via the modulation of the endocannabinoid system, since it is antagonized by a cannabinoid CB1 receptor antagonist. Thus, the combination of lower doses of indomethacin, or other NSAIDs, and minocycline could be useful in the management of established CIPN, a condition where there is a dearth of effective drugs to manage.

## Methods

### Animals

Female and male BALB/c mice (8 to 12 weeks old; 20–30 g, n = 136 for female mice and 70 for male mice) supplied by the Animal Resources Center at the Health Sciences Center (HSC), Kuwait University, Kuwait, were used in this study in order to address the issues of gender response to drug treatment. Animals were kept in temperature controlled (24 ± 1 °C) rooms with food and water ad libitum. All experiments were performed during the same period of the day (8:00 AM to 4:00 PM) to exclude diurnal variations in pharmacological effects. The animals were handled in compliance with European Communities Council Directive 86/609 for the care of laboratory animals and ethical guidelines for research in experimental pain with conscious animals^34^. All procedures were approved by the Ethical Committee for the use of Laboratory Animals in Teaching and Research, HSC, Kuwait University.

### Administration of paclitaxel to induce neuropathic pain

A solution made up of 50% Cremophor EL and 50% absolute ethanol was used to dissolve paclitaxel (Tocris, Bristol, UK) to a concentration of 6 mg/ml and stored at −20 °C, for a maximum of 14 days. The 6 mg/ml paclitaxel solution was further diluted in normal saline (NaCl 0.9%), to a final concentration of 0.2 mg/ml just before administration to mice. The vehicle for paclitaxel was diluted at the time of injection with normal saline in the same proportion as the paclitaxel solution. Paclitaxel 2 mg/kg or its vehicle were administered to mice intraperitoneally (i.p.), in a volume of 10 ml/kg, once per day for 5 consecutive days; the cumulative dose of paclitaxel was 10 mg/kg. We have previously observed thermal hyperalgesia in mice using this treatment regimen^18^.

### Drug administration

Indomethacin (Sigma-Aldrich, St Louis, MO, USA) was dissolved in peanut oil; minocycline (Sigma-Aldrich, St Louis, MO, USA) in phosphate buffered saline and AM 251 (Tocris, Bristol, UK) in normal saline containing 5% Tween 80 and 5% propylene glycol. The drugs were freshly prepared before administration and administered i.p. to mice at a volume of 5 ml/kg body mass. For the evaluation of coadministration, mice received separate i.p. injections at the same time: indomethacin + vehicle for minocycline + vehicle for AM 251, minocycline + vehicle for indomethacin + vehicle for AM 251, AM 251 + vehicle for minocycline + vehicle for indomethacin, indomethacin + minocycline + vehicle for AM 251 and indomethacin + minocycline + AM 251. The drugs were administered to paclitaxel treated mice at 7 days after first administration of paclitaxel, when mice had developed thermal hyperalgesia as previously described^18^.

### Assessment of thermal nociception

Reaction latencies of mice to hot plate (Panlab SL, Barcelona, Spain) at 55 ± 1 °C in the form of the first sign of nociception, paw licking, flinching or jump response to avoid the heat were measured, as described before^18^, before (baseline latency), at day 7 after first injection of paclitaxel and at various times after drug treatment. A cut-off period of 20 seconds was maintained to avoid damage to the paws.

### Statistical analyses

Statistical analyses were performed using unpaired Student’s t test, one-way analysis of variance (ANOVA) followed by Dunnett’s multiple comparison test, or two-way repeated measures ANOVA followed by Bonferroni posttests. The differences were considered significant at p < 0.05. The results in the text and figures are expressed as the means ± S.E.M.

## Additional Information

**How to cite this article**: Parvathy, S. S. and Masocha, W. Coadministration of indomethacin and minocycline attenuates established paclitaxel-induced neuropathic thermal hyperalgesia: Involvement of cannabinoid CB_1_ receptors. *Sci. Rep.* **5**, 10541; doi: 10.1038/srep10541 (2015).
